# Editorial: Innovation and trends in the global food systems, dietary patterns and healthy sustainable lifestyle in the digital age

**DOI:** 10.3389/fnut.2023.1211186

**Published:** 2023-07-24

**Authors:** Maha Hoteit, Radwan Qasrawi, Haleama Al Sabbah, Reema Tayyem

**Affiliations:** ^1^Food Sciences Unit, National Council for Scientific Research Lebanon (CNRS-L), Beirut, Lebanon; ^2^Department of Computer Science, Al-Quds University, Jerusalem, Palestine; ^3^Department of Computer Engineering, Istinye University, Istanbul, Türkiye; ^4^Department of Public Health, College of Health Sciences, Abu Dhabi University, Abu Dhabi, United Arab Emirates; ^5^Department of Human Nutrition, College of Health Sciences, Qatar University, Doha, Qatar

**Keywords:** editorial, innovation, food system, dietary patterns, sustainability

The transformation of food systems to address healthy nutrition, food insecurity, and public health issues is a global concern. In today's world, where there is a growing dependence on processed foods, fast food, edible oils, sugar-sweetened beverages, and sedentary lifestyles, the importance of food security and nutrition systems cannot be overstated. These factors have a direct impact on human wellbeing and global stability.

The increasing prevalence of non-communicable diseases such as obesity, diabetes, hypertension, and cardiovascular diseases is a clear indication of the impact of lifestyle changes which are being observed worldwide (1). Although food security has improved in developed countries, many countries, particularly low-to middle-income countries (LMIC), suffer from significant food insecurity challenges (2). In addition, food production, accessibility, and availability have been further impacted due to the COVID-19 outbreak, causing growing global concerns regarding food security, especially within the most vulnerable communities (3). Due to this situation and in this dynamic context, both technology actors and the consumers they cater to occupy a crucial position in the food system. They have the potential to make choices that can address the challenges and opportunities for enhancing sustainable outcomes in the food system. According to the literature, the integration of artificial intelligence, the deployment of data science techniques, and information and communication technology (ICT) applications have introduced innovative methods in understanding the trends in food systems, dietary patterns, and healthy sustainable lifestyles at the global level (4).

The main objective of this Research Topic is to gather papers that enhance our understanding of the intersections between food security, nutrition, and technological innovation through the utilization of artificial intelligence, data science, and ICT. These papers aim to provide comprehensive insights into their impact on human health and to analyze global trends before and after the COVID-19 pandemic. The special e-collection comprises 15 papers that cover the aforementioned areas. The most extensively examined aspect in the Eastern Mediterranean Region (EMR), both before and during the COVID-19 pandemic, has been the nutrition-related factors and dietary intake of its populations.

Data from Al-Jawaldeh and Abbass show that the prominent nutrition-related non-communicable diseases (NCDs) risk factors in EMR include obesity, hypertension, high fasting plasma glucose, and upregulated unhealthy diet consumption, which can lead to nutrition inadequacies, including anemia. According to the authors, these risk factors, even if treated, are often poorly controlled. Therefore, it is imperative to adopt healthy dietary habits and ensure adequate physical activity to counter their presence and prevent their onset. To tackle and manage obesity, Jabbour et al. published a meta-analysis (NMA) on the current Research Topic, which compared the effects of different diets [moderate macronutrients (MMs), low fat/high carbohydrate (LFHC), high fat/low carbohydrate (HFLC), and usual diet (UD)] on weight, body mass index (BMI), and waist circumference (WC) changes at ≥12 months. The authors show that dietary interventions extending over ≥12 months are superior to UD in inducing weight, BMI, and WC loss. HFLC might be associated with a slightly higher weight loss compared with MM diets. In contrast, this Research Topic also addresses nutritional inadequacy, specifically anemia, by using a novel analytical method that was implemented to classify nutritional anemia using the cluster analysis approach. In this study, Qasrawi and Al-Halawa conducted the Classification and Regression Tree (CRT) model to study the association between hemoglobin clusters and vitamin B12, folate, and iron intakes, sociodemographic variables, and health-related risk factors, accounting for grade and age. The study findings indicated that vitamin B12, iron, and folate intakes are important factors related to anemia. In girls, anemia was associated with age, locality, food consumption patterns, and physical activity levels, whereas, iron and folate intakes were significant factors related to anemia in boys associated with the place of residence and the educational level of their mothers. According to the authors, the deployment of clustering and classification techniques for identifying the association between anemia and nutritional factors might facilitate the development of nutritional anemia prevention and intervention programs that will improve the health and wellbeing of schoolchildren. Outside the EMR, in Chile, a study conducted by Schnettler et al., showed that there were positive associations between mothers' modeling and adolescents' satisfaction with food-related life (SWFoL) between mothers' diet quality and fathers'; and between mothers' modeling and fathers' SWFoL via the fathers' diet quality. Parents' modeling can improve the three family members' diet quality, while mothers' modeling and diet quality has been shown to improve fathers' and adolescents' SWFoL. Knowing that good dietary patterns start from the prenatal period, Abi Khalil et al. investigate the feeding patterns of women and their off-springs in Lebanon. Data from Abi Khalil et al. demonstrate low rates of exclusive breastfeeding (EBF) and continuous breastfeeding (CBF), high prevalence of exclusive bottle feeding (EBOT), and early introduction of complementary foods among children ages 0–59 months. Furthermore, for children ages 6–59 months, there was poor mother-child dietary diversity and a high prevalence of overweight and stunted children in the main two Lebanese provinces.

The Mediterranean Diet (MedDiet) represents a healthy dietary pattern in the context of healthy lifestyle habits. Epidemiological studies have shown that a higher degree of adherence to the MedDiet pattern is associated with reduced mortality concerning deaths due to non-communicable diseases. This is favored by the adoption of this dietary pattern. To investigate the current situation concerning adherence to the Mediterranean diet in Lebanon and Syria, Karam et al. showed that the Lebanese participants, especially men and those who are aged between 64 and 67, had higher adherence than their counterparts. It is essential to invest in the education and behavior of healthcare professionals as it can significantly impact the dietary habits of their patients, ultimately aiding in the promotion of a healthy Mediterranean diet and the prevention of unhealthy eating patterns.

This was identified by Hoteit, Mohsen et al., who show that the risk of eating disorders was prevalent in 22.5% of healthcare practitioners in Lebanon. The highest proportion of high-risk participants were participants studying and practicing nutrition (40.9%), outracing their counterparts in nursing (18.7%), medicine (17.8%), pharmacy (17.7%), and midwifery (4.9%), sciences (*p* = 0.02).

The impact of the COVID-19 pandemic on dietary diversity and intake has also been widely discussed. According to Hoteit, Mortada et al.'s data, the fragility of the EMR food system is presenting significant obstacles to maintaining a healthy and sustainable lifestyle. While the aggressive containment strategy from the COVID-19 pandemic was essential for most countries in the EMR to help prevent the spread, it came at a high nutritional cost, driving poor dietary diversity. This situation affects maternal diets as well. This was identified in a survey that was carried out among a convenient sample of 1,939 pregnant women from five Arab countries by Hoteit, Hoteit et al., who found an increment in the consumption of cereals, fruit, vegetables, dairy products, meats, and nuts that occurred during the COVID-19 pandemic. In this survey, the daily consumption of almost all food groups was lower than the USDA's daily recommendations, except for fruits intake, which was higher than the daily standard. Demonstrated poor adherence to prenatal USDA dietary guidelines by Arab pregnant women can lead to numerous deficiencies and health risks among their offspring. According to the authors, the findings emphasize the need for nutritional education and intervention during prenatal visits. Similarly, Papazian et al. also found that dietary patterns among pregnant women should be monitored regularly. In Lebanon, pregnant women with the lowest pre-gestational body mass index and higher gestational age tended to follow a diet called “Neo-Mediterranean,” which is mainly composed of protein-rich foods such as poultry, fish, eggs, and dairy products.

Tayyem et al.'s survey showed that postpartum women exhibit poor adherence to the USDA's recommended amounts for dietary intake and the five food groups, with their dietary intake falling below the recommended levels.

During the COVID-19 pandemic, while many people were forced to stay home, scholars began investigating the impact of online food delivery services on health and wellbeing. These services may affect the achievement of the United Nations 2030 Sustainable Development Goals (SDGs). According to Jia et al.'s research, online food delivery services may pose a threat to several SDGs, including those addressing good health and wellbeing, responsible consumption and production, climate action, and decent work and economic growth.

The scholars in this study proposed a research and policy agenda that is aligned with entry points within a systems approach identified by the World Health Organization, which are the food industry reforms, synergized public health messaging, and the continuous monitoring of the growing impact of online food delivery.

In summary, the results of the above-mentioned studies and reviews represent an enormous amount of new relevant data on obesity management, food insecurity, dietary patterns among all age categories, novel cluster models, and the impact of the COVID-19 pandemic on all the listed topics. Despite all the existing literature and evidence related to these extremely important topics, the papers published in this e-book clearly show that there are still many aspects to be clarified and understood in the fascinating world of dietary diversity, food insecurity, dietary intake, and the management of non-communicable diseases. Upon reading this book, readers will gain clarity on various topics and develop a stronger conviction regarding the significance of studying nutrition systems, food security, and the roles of technological advances, especially in LMIC. Understanding these factors is crucial for comprehending the food transition and population health. Such understanding is imperative to prioritize equity, sustainability, and health as priorities in food provision and consumption in LMIC (5).

## Author contributions

MH, RQ, HA, and RT: conceptualization, validation, supervision and editorial administration, methodology, and resources. MH and RT: formal analysis, data curation, and writing—original draft preparation. All authors contributed to the editorial and approved the submitted version.
